# Pregnancy and mental health in times of COVID-19

**DOI:** 10.1192/j.eurpsy.2021.723

**Published:** 2021-08-13

**Authors:** M.O. Solis, M. ValverDe Barea, S. Jimenez Fernandez, S.S. Sánchez Rus

**Affiliations:** Jaén, Complejo Hospitalario Jaén, Jaén, Spain

**Keywords:** COVID-19, pregnancy, Beck Inventory, mental health

## Abstract

**Introduction:**

The new coronavirus (COVID-19) is being a threat to global health. Pregnancy is considered a state of vulnerability to mental health and can be even greater if they are facing the current pandemia.

**Objectives:**

Within this framework, we wanted to inquire about the state of mental health, and more specifically, about depression, during pregnancy during pandemia COVID-19 and their opinion of the health team∙s professionals that controls pregnancy and how they are involved in the assessment of their mental health status. Also know her fears and uncertainties about the virus and its possible consequences (complications during pregnancy, childbirth or confinement at home, possible contact with COVID-19 positive patients).

**Methods:**

A cross-sectional study was carried out that includes 73 pregnant women from Spain, during September 2020, through an anonymous, voluntary and multiple response type online survey which included questions about socio demographic aspects and the Beck Depression Inventory.

**Results:**

The average age was 32 years. 90.41% were with a partner or married. The results of Beck’s questionnaire: 24.65% have moderate/severe depression. 25.65% had or had thought about consulting a mental health professional, 90.41% considered that professionals had not asked about their mental health during pregnancy. 98.89% reported fear of becoming infected and having to confine themselves at home and 97.26% reported fear of get COVID and affect the health of the unborn baby.

**Conclusions:**

COVID-19 represents a huge challenge for pregnant women’s mental health. The Development and implementation of mental health service, skilled and aware of this area is crucial, for this vulnerable population.

